# Single Bursts of Individual Granule Cells Functionally Rearrange Feedforward Inhibition

**DOI:** 10.1523/JNEUROSCI.1595-17.2018

**Published:** 2018-02-14

**Authors:** Máté Neubrandt, Viktor János Oláh, János Brunner, Endre Levente Marosi, Ivan Soltesz, János Szabadics

**Affiliations:** ^1^Institute of Experimental Medicine, Hungarian Academy of Sciences, Budapest, 1083, Hungary,; ^2^Department of Neurosurgery, and; ^3^Stanford Neurosciences Institute, Stanford University, Stanford, California 94305

**Keywords:** burst firing, feedforward inhibition, hippocampus

## Abstract

The sparse single-spike activity of dentate gyrus granule cells (DG GCs) is punctuated by occasional brief bursts of 3–7 action potentials. It is well-known that such presynaptic bursts in individual mossy fibers (MFs; axons of granule cells) are often able to discharge postsynaptic CA3 pyramidal cells due to powerful short-term facilitation. However, what happens in the CA3 network after the passage of a brief MF burst, before the arrival of the next burst or solitary spike, is not understood. Because MFs innervate significantly more CA3 interneurons than pyramidal cells, we focused on unitary MF responses in identified interneurons in the seconds-long postburst period, using paired recordings in rat hippocampal slices. Single bursts as short as 5 spikes in <30 ms in individual presynaptic MFs caused a sustained, large increase (tripling) in the amplitude of the unitary MF-EPSCs for several seconds in ivy, axo-axonic/chandelier and basket interneurons. The postburst unitary MF-EPSCs in these feedforward interneurons reached amplitudes that were even larger than the MF-EPSCs during the bursts in the same cells. In contrast, no comparable postburst enhancement of MF-EPSCs could be observed in pyramidal cells or nonfeedforward interneurons. The robust postburst increase in MF-EPSCs in feedforward interneurons was associated with significant shortening of the unitary synaptic delay and large downstream increases in disynaptic IPSCs in pyramidal cells. These results reveal a new cell type-specific plasticity that enables even solitary brief bursts in single GCs to powerfully enhance inhibition at the DG-CA3 interface in the seconds-long time-scales of interburst intervals.

**SIGNIFICANCE STATEMENT** The hippocampal formation is a brain region that plays key roles in spatial navigation and learning and memory. The first stage of information processing occurs in the dentate gyrus, where principal cells are remarkably quiet, discharging low-frequency single action potentials interspersed with occasional brief bursts of spikes. Such bursts, in particular, have attracted a lot of attention because they appear to be critical for efficient coding, storage, and recall of information. We show that single bursts of a few spikes in individual granule cells result in seconds-long potentiation of excitatory inputs to downstream interneurons. Thus, while it has been known that bursts powerfully discharge (“detonate”) hippocampal excitatory cells, this study clarifies that they also regulate inhibition during the interburst intervals.

## Introduction

Bursts of high-frequency action potentials (APs) are distinct neuronal signals generated by nonlinear dendritic processes in response to coincident inputs that result in a reliably transmitted output (Lisman, 1997; Harris et al., 2001; Xu et al., 2012; Apostolides et al., 2016). The granule cells of the dentate gyrus (DG GCs) are among the most quiescent neurons in the cortical mantle with an unusually low overall firing rate (<1 Hz *in vivo*), but they are also capable of discharging brief bursts of 3–7 APs within tens of milliseconds (100–200 Hz), separated by long silent periods (2–15 s). These bursts occur during distinct behaviors (Buzsáki et al., 1983; Jung and McNaughton, 1993; Henze et al., 2002; Pernía-Andrade and Jonas, 2014; Diamantaki et al., 2016; Danielson et al., 2017; GoodSmith et al., 2017; Senzai and Buzsáki, 2017) and are channeled to the CA3 region by the axons of the DG GCs, the mossy fibers (MF), where each MF innervates only 11–18 CA3 pyramidal cells but several times more GABAergic cells (40–50 cells) (Acsády et al., 1998).

The well-studied response of postsynaptic CA3 pyramidal cells during MF bursts is characterized by strong short-term facilitation that enables the MF-generated EPSPs to reach firing threshold and almost invariably discharge the pyramidal cells, a property referred to as “conditional detonation” (conditional in the sense that if there is a presynaptic burst, then there is pyramidal cell firing) (Salin et al., 1996; Tóth et al., 2000; Gundlfinger et al., 2010; Chamberland et al., 2014; Vyleta et al., 2016). The MFs innervate at least four distinct types of GABAergic interneurons in the CA3, with the MF-interneuron synaptic connections displaying cell type-specific properties and short-term plasticity rules that result in a weaker excitation of the MF-CA3 GABAergic interface than the conditional detonation displayed by the CA3 pyramidal cells (Henze et al., 2002; Lawrence and McBain, 2003; Mori et al., 2004; Nicoll and Schmitz, 2005; Szabadics and Soltesz, 2009; Zucca et al., 2017). In contrast to the availability of cell type-specific information about the postsynaptic events triggered by MF activity during the burst in CA3 pyramidal cells and distinct interneuron types, virtually nothing is known about how physiologically realistic (i.e., brief, single) bursts of APs in individual MFs shape the MF-CA3 GABAergic network in the seconds-long interburst period, after the occurrence of one burst and before the arrival of the next. Indeed, while previous studies have shown that changes in the MF-evoked excitation of CA3 inhibitory cells can persist for tens of seconds or even minutes after prolonged high-frequency stimulation (300 APs at 30–40 Hz) (Alle et al., 2001; Mori et al., 2007), the effect of shorter physiological MF burst activity on CA3 interneurons remains unknown.

Here we combined paired recordings from the presynaptic GCs that reside in the CA3, or directly from MF boutons of DG GCs, with recordings from postsynaptic, *post hoc* identified CA3 cells to study how single, brief bursts of APs in individual MFs modify the unitary MF responses in the postburst period. The results show that even single bursts formed by a few spikes occurring in <30 ms, representing physiologically relevant bursts that actually occur *in vivo*, cause a sustained (up to 8 s) tripling of the amplitude of the unitary MF-EPSCs in feedforward interneurons (FF-INs), including axo-axonic/chandelier cells, two types of basket cells and ivy cells. The tripling of the MF-EPSCs after a single brief burst activity was specific to FF-INs, and no comparable postburst potentiation could be observed in either CA3 pyramidal cells or extrahippocampally projecting GABAergic neurons. Furthermore, the postburst enhancement of the MF-EPSCs in FF-INs was strong enough to cause large, sustained increases in disynaptic IPSCs in randomly sampled CA3 pyramidal cells.

These results reveal a novel type of MF plasticity that makes it possible for even solitary, brief bursts of APs in single GCs to enhance CA3 inhibition at time-scales that are commensurate with the physiological interburst intervals.

## Materials and Methods

### 

#### 

##### Slice preparation, recording conditions, and processing for anatomy.

For acute slice preparations, adolescent Wistar rats (postnatal day 21–45, both sexes) were deeply anesthetized with isoflurane (in accordance with the ethical guidelines of the Institute of Experimental Medicine Protection of Research Subjects Committee; 22.1/1760/003/2009), and 350 μm slices were cut in ice-cold ACSF containing the following (in mm): 85 NaCl, 75 sucrose, 2.5 KCl, 25 glucose, 1.25 NaH_2_PO_4_, 4 MgCl_2_, 0.5 CaCl_2_, and 24 NaHCO_3_. The slices were cut from the dorsal and medial part of the hippocampus using a standard vibratome (Leica VT1200S, 0.08 mm/s speed and 1 mm amplitude). The slice orientation was, as in Szabadics and Soltesz (2009), intended to be parallel to the MFs and many CA3 dendrites to maximize the preservation of MF connections to the CA3. The slices were incubated at 32°C for 60 min after sectioning and were then stored at room temperature until they were used for recordings within 10 h. The cells were visualized with an upright microscope (Eclipse FN-1; Nikon) with infrared (900 nm) Nomarksi differential interference contrast optics (40× 0.8 NA water-immersion objective). The standard recording ACSF was composed of the following (in mm): 126 NaCl, 2.5 KCl, 26 NaHCO_3_, 2 CaCl_2_ (unless stated otherwise), 2 MgCl_2_, 1.25 NaH_2_PO_4_, and 10 glucose at 35 ± 0.5°C.

Electrophysiological recordings were obtained with MultiClamp 700B amplifiers (Molecular Devices) and pClamp10 software (sampling rate 50 kHz). Postsynaptic cells were voltage-clamped at −70 mV (low-pass filtered at 4–6 kHz). Series resistance (5–30 mΩ) was monitored by the capacitive artifact in response to a 5 mV step in each trace. Presynaptic CA3 GCs were recorded in current clamp (low-pass filtered at 10–20 kHz), whereas large MF terminals were preferentially assessed in cell-attached configuration with pipettes containing intracellular solution (Szabadics and Soltesz, 2009; Vyleta and Jonas, 2014); some giant MF terminals were recorded in current-clamp mode. The pipette capacitance was greatly reduced, but not fully neutralized, in the bridge-balance-compensated current-clamp recordings. Recording pipettes were pulled from either thin- or thick-walled (1.12 or 0.86 mm inner diameter, 1.5 outer diameter) borosilicate glass capillaries; the pipette resistance ranged between 3 and 4.5 mΩ for the somatic recordings or between 10 and 12 mΩ for the axonal recordings.

Three different intracellular solutions were used. Under standard recording conditions, pairs were recorded in an intracellular solution containing the following (in mm): 90 K-gluconate, 43.5 KCl, 1.8 NaCl, 1.7 MgCl_2_, 0.05 EGTA, 10 HEPES, 2 Mg-ATP, 0.4 Na_2_-GTP, 10 phosphocreatine-disodium, and 8 biocytin (pH 7.25). In one set of experiments (see Fig. 3*B–D*), presynaptic recordings were performed with a modified version of the first intracellular solution (40 mm KCl was substituted for CsCl) to increase the release probability of the MF-spiny lucidum cell (SLC) connections. The third intracellular solution was optimal for assessing the disynaptic connections because of the large difference between the reversal potentials of the GABAergic and glutamatergic events; specifically, the postsynaptic pyramidal cells were patched with a low [Cl^−^] intracellular solution composed of the following (in mm): 133.5 K-gluconate, 1.8 NaCl, 1.7 MgCl_2_, 0.05 EGTA, 10 HEPES, 2 Mg-ATP, 0.4 Na_2_-GTP, 10 phosphocreatine-disodium, and 8 biocytin (pH 7.25). The chemicals for the intracellular and extracellular solutions were purchased from Sigma-Aldrich, and the various pharmacons were purchased from Tocris Bioscience. DCG IV (1 μm), MSOP (150 μm), and SR 95531 (gabazine, 5 μm) were dissolved in ACSF. Phorbol 12,13-dibutyrate (PDBu, 1 μm), Go 6976 (250 nm), GF 109203X (1 μm), calphostin C (1 μm), U 73122 (2.5 μm), BINA (5 μm), AMN 082 (1 μm), and KT5720 (200 nm) were dissolved first in DMSO and diluted at least 2000 times in ACSF. EGTA (0.5–2.5 mm), PKC19-36 (100 μm), and PKA inhibitory fragment 6–22 amide (PKI, 2.5 μm) were added to the intracellular solution. In the case of GF109203X, Go6976, and KT5720, the slices were also preincubated before recordings. Otherwise, the drugs were added to the perfused ACSF.

**Figure 1.**
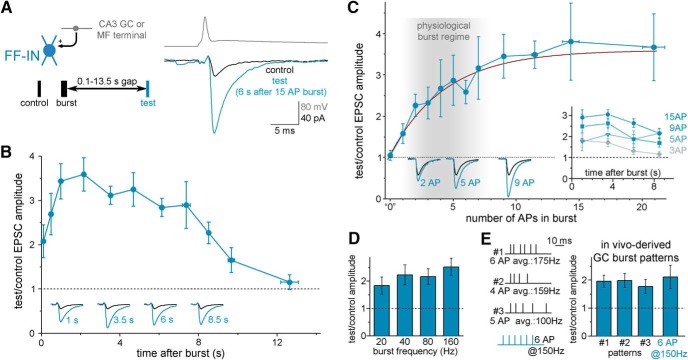
Single, brief MF bursts potentiate monosynaptic MF-EPSCs in CA3 FF-INs for several seconds. ***A***, Schematic representation of the recording configuration for unitary MF responses in CA3 neurons. Presynaptic patch-clamp recording was obtained from a single giant MF terminal (most of these originate from DG GCs; Fig. 1-1) or a single CA3 GC (somatic recording; these cells are rarer but easier to record than the MF terminals); the postsynaptic cell was an FF-IN. MF output was first measured with a control stimulus, followed by a single 2–20 AP burst (150 Hz); after various time delays (0.1–13.5 s), which approximated the typical physiological inactivity periods in GCs *in vivo*, a test response was evoked. Right, Example traces of single-AP-evoked control and test EPSCs in an identified FF-IN (the illustrated recordings were from an IvyC; for the anatomy of the presynaptic and postsynaptic cells, see Fig. 1-1). In most cases, control and test pulses contained 3 APs at 20 Hz to gain insight into possible changes in short-term plasticity after the bursts; plots in this and subsequent figures show responses to the first APs in the triplet, except in Figure 4*C*, *D*. ***B***, Time course of the relative amplification of monosynaptic MF-EPSCs after single 15 AP presynaptic bursts in the same individual MF. Relative postburst EPSC amplitudes are shown (i.e., control relative amplitudes are 1, dashed line). The *x* axis indicates the time of the test pulse after the burst. The graph includes all data points (*n* = 280, first APs) from identified FF-IN pairs (*n* = 78 pairs; including IvyCs: *n* = 55 pairs; AACs: *n* = 10; PV+BC: *n* = 5; CCK+IN, *n* = 8; for separate analysis of the burst-induced amplification in different postsynaptic cell types, see Figs. 1-1, 1-2, 1-3, and 1-4). Insets, Example test responses in blue at different postburst delays; same postsynaptic IvyC as in ***A***; control traces are black. ***C***, Dependence of the postburst potentiation on AP numbers within the burst (from *n* = 99 data points from *n* = 28 pairs). Brown curve indicates the exponential fit of the data (*R*^2^ = 0.966). Gray area represents the typical range of GC bursts *in vivo*. Insets, Example test and control traces and the time course of the changes of the responses after 3, 5, 9, and 15 AP bursts. ***D***, Effects of single presynaptic bursts consisting of 8 APs at 20, 40, 80, and 150 Hz, on the same MF-FF-IN pairs (*n* = 7). ***E***, Effects of single bursts, whose patterns were obtained from *in vivo* recorded identified GCs: #1, 6 APs with 3.7, 7.5, 4.54, 6.98, and 5.88 ms interspike intervals (Henze et al., 2002); #2, 4 APs with 4, 6, and 9 ms interspike intervals (Pernía-Andrade and Jonas, 2014); #3, 5 APs with 6, 8, 11, and 15 ms interspike intervals (Diamantaki et al., 2016); for comparison, the effect of a 6 AP 150 Hz burst is also shown. The effects were measured with each realistic burst protocol in the same pairs (*n* = 8 pairs, 3.6 s after burst).

After the recordings, the slices were fixed for 1 d in 0.1 m phosphate buffer containing 2% PFA and 0.1% picric acid at 4°C. After fixation, the slices were resectioned at 60 μm. For immunocytochemistry, the sections were incubated with one or two of the following primary antibodies against parvalbumin (PV; PV25 and PV27, 1:1000, polyclonal rabbit, Swant), SATB1 (sc-5989, 1:400, polyclonal goat, Santa Cruz Biotechnology), cholecystokinin (CCK; C2581, 1:1000, polyclonal rabbit, Sigma-Aldrich), somatostatin (MAB354, 1:500, monoclonal rat, Millipore Bioscience Research Reagents), or neuronal nitric oxide synthase (N2280, 1:500 mouse, Sigma-Aldrich) overnight in 0.5% Triton X-100 and 2% normal goat serum or horse serum containing TBS buffer at 4°C. The immunoreactions were visualized with AlexaFluor-488- or AlexaFluor-594-conjugated secondary goat or donkey antibodies (1:500; Invitrogen) against rabbit, goat, mouse, and rat IgGs, and biocytin staining was revealed using AlexaFluor-350- or AlexaFluor-488-conjugated streptavidin. The recorded cells were analyzed on epifluorescence microscope (DM2500, Leica). Multiple image stacks were acquired from a 60-μm-thick slice to visualize the axonal and dendritic arbors. The maximum-intensity-projected black-and-white fluorescence images were inverted for better visualization of a large part of the dendritic and axonal arborization. One recorded pair (Fig. 3-1) and a few other test samples were processed for DAB staining (Szabadics and Soltesz, 2009). Because DAB staining did not provide a better substrate for anatomical identification than fluorescent labeling, the other analyses were completed using the latter, methodologically simpler staining method.

##### Cell types.

A total of 223 monosynaptic pairs were included in this work. However, the majority of the analysis included only those pairs where the postsynaptic cell was identified as an FF-IN (*n* = 116 pairs, including IvyC, AAC, PV+BC, and CCK+IN types), SLC (*n* = 25 pairs), or pyramidal cell (*n* = 12 pairs). The single MF source for monosynaptic connections was either a giant MF terminal in the stratum lucidum (*n* = 7) or a CA3 GC (*n* = 216, including divergent connections from the same presynaptic source). The giant MF terminals specifically innervate pyramidal cells, whereas GABAergic cells receive MF inputs from small en passant or filopodial boutons. Thus, giant MF terminal recordings in whole-cell mode are able to maintain stable release onto GABAergic cells (Szabadics and Soltesz, 2009). The other presynaptic MF source was CA3 GCs, which provide a reliable, easily accessible, and stable model for studying single MF inputs using standard whole-cell paired recordings (Szabadics et al., 2010). CA3 GCs were identified based on the location of their soma, typical GC firing pattern, and polarized morphology; straight spiny dendrites were mostly oriented toward the stratum lacunosum-moleculare, and the axons with large MF terminals were in stratum lucidum or stratum pyramidale. The CA3 GCs within this sample showed similar cellular properties as fully mature GCs (Brunner et al., 2014).

Postsynaptic cells were classified based on multiple criteria. IvyCs (*n* = 87 pairs) were identified by their characteristic firing pattern (late firing, large and slow after hyperpolarization), short dendrites, and dense axon arborization mainly in the strata radiatum and oriens. Nine of the 14 tested IvyCs were neuronal nitric oxide synthase-positive, and none of the tested IvyCs was positive for CCK or somatostatin (*n* = 20 and *n* = 20, respectively). PV+BCs (*n* = 5) were identified based on their fast-spiking activity and axons that specifically targeted the stratum pyramidale. All tested PV+BCs were immunopositive for PV (*n* = 4) and SATB1 (*n* = 2) (Viney et al., 2013). AACs (*n* = 10) were identified based on their fast spiking activity and characteristic axons apparently outlining the axon initial segments at the border of strata pyramidale and oriens, as well as the presence of PV (8 of the 9 tested cells) and lack of SATB1 immunopositivity (*n* = 6). Regular-spiking and CCK-expressing neurons (*n* = 11) were classified as CCK+INs, including both basket cells and dendrite targeting cells. SLCs (*n* = 25) were identified based on their densely spiny dendrites, which were usually restricted to the strata lucidum and pyramidale and somatostatin immunopositivity (15 positive of 19 tested) and lack of CCK expression (*n* = 13 tested). Pyramidal cells were targeted in the stratum pyramidale and were identified based on their typical firing pattern and morphology, including thick, densely spiny dendrites and complex spines in the stratum lucidum.

Disynaptic IPSCs (diIPSCs) were assessed in paired recordings of MF terminals or CA3 GCs and pyramidal cells in a separate set of experiments using less Cl^−^-containing intracellular solution for the unequivocal distinction of EPSCs and IPSCs. The membrane potential was held usually at −50 mV (above the reversal potential of Cl^−^). diEPSCs were observed in postsynaptic interneurons. The onset delays of the diEPSCs were clearly distinguishable from monosynaptic EPSCs (see Fig. 7*E*). The excitatory nature of the events was confirmed by depolarizing the postsynaptic cells close to the reversal potential of Cl^−^ or by gabazine wash in. diIPSCs and diEPSCs were analyzed in a predefined time window (1.5–6.5 and 2–7 ms, respectively, from the peak of the presynaptic AP), in which all events were counted, potentially including a few spontaneous events originating from other presynaptic sources.

##### Experimental design and statistical analysis.

The postsynaptic responses were tested in each recorded pair with multiple (usually 3 or 4) different protocols, varying either the delay between the burst and the test pulses (between 0.1 and 13.5 s) or the number and pattern of APs within the burst (1–20 APs), and the measurements were considered to be independent data points for the analysis. Each trace included one control, one burst, and one test stimuli. Control stimuli (usually 3 APs at 20 Hz) were followed by a single burst within 200 ms. For investigating the temporal profile of the effects of single presynaptic bursts, the only difference between consecutive traces was the gap between the burst and test stimuli. The next trace was recorded after allowing for the responses to recover to control levels (at least 60 s). For investigating the effects of various presynaptic burst patterns, we followed the same strategy as above, except the timing between the burst and test pulses were constant in individual protocols and only the pattern was varied (i.e., number or frequency of APs within the burst). After testing each different protocol once, we repeated them consecutively to collect the sufficient numbers of traces. Thus, the different protocols gave directly comparable results and included reliable internal controls (the control responses before burst). Only those consecutive traces were considered for analysis in which the amplitudes, short-term plasticity and kinetics of the control responses were stable (i.e., without run down or long-term changes).

EPSC peak amplitudes were measured relative to a preceding baseline period on average traces. Minimum 3, but usually 5–10 traces were averaged for every protocol. For control responses (i.e., before bursts), all traces were averaged, thus, the same control was considered for 3–4 different postburst data points within each pair. Paired pulse ratios (PPRs) were calculated from the average traces by dividing the average of the second and the third EPSC amplitudes with the first EPSC amplitude of the 20 Hz control and test responses. Synaptic delays were measured at individual events from the peak of the presynaptic AP to the onset of the events. Data are presented as mean ± SE. Statistical tests are indicated within the text. Normality of distributions was tested with the Shapiro–Wilks test. One-sample or two-sample unpaired Student's *t* test and paired Student's *t* test are indicated as *t* test and paired *t* test within the text. Degrees of freedom are indicated after each *t* (for *t* tests) or *F* (for ANOVA) values in parentheses.

## Results

### Paired recordings to test the effects of physiologically relevant burst activity in single GCs on identified postsynaptic CA3 cells

We tested the postburst consequences of single MF bursts in various types of postsynaptic CA3 neurons using unitary responses in paired patch-clamp recordings (for the experimental arrangement, see Fig. 1*A*, left). For the presynaptic side, we obtained precise control of the synaptic outputs of single presynaptic MFs by using two different approaches. First, direct intracellular or cell-attached recordings from MF terminals allowed us to assess the properties of unitary EPSCs from individual DG GCs to CA3 neurons (Szabadics and Soltesz, 2009). Second, somatic recordings from ectopic GCs (CA3 GCs), whose synaptic properties are indistinguishable from those of DG GCs (Szabadics et al., 2010), allowed for a reliable comparison of MF-to-CA3 neuron EPSCs across multiple protocols, which require long-term recordings. The results from the two types of presynaptic MF recordings were comparable and were therefore grouped together for analysis (for direct comparison of the effects of the two MF sources, see below). The postburst potentiation was primarily assessed by comparing the relative amplitudes of preburst control and postburst test MF-EPSCs (e.g.; Fig. 1*B*, *y* axis) with the test responses following the bursts within hundreds of milliseconds to several seconds (*x* axis in Fig. 1*B*).

10.1523/JNEUROSCI.1595-17.2018.f1-1 Figure 1-1 Postsynaptic Ivy cells (IvyC): Morphological identification, comparison with the pooled data from all FF-INs and representative MF terminal (presynaptic DG GC) to IvyC and CA3 GC to IvyC pairs. ***A*,** Upper panel: firing pattern of the presynaptic MF terminal; lower: example traces show the presynaptic AP in the MF terminal and the EPSC response in the IvyC. ***B*,** Back-labeled parent GC soma (B_1_) in the DG following the recording of the presynaptic MF terminal in the CA3; and MF terminals (B_2_) along the recorded axon. ***C*,** Axon morphology (C_1_) and immunohistochemical testing (C_2_) of the postsynaptic IvyC in the *stratum radiatum* of the CA3. The inset shows the firing pattern of the IvyC. ***D*,** Control and post-burst test MF-EPSCs from the example MF bouton-IvyC pair, 8.4 seconds after the burst (15 AP at 150 Hz). ***E*,** Comparison of the amplification of the MF responses from IvyC pairs (n = 55) with the pooled FF-IN data (see Fig. 1B). The insets show the control (before burst) and four different delays after the single bursts from the pair shown in panel D. ***F-G***, Identification of the presynaptic CA3 GC and the postsynaptic IvyC shown in **Fig. 1A-B. *F*,** The presynaptic CA3 GC and the postsynaptic IvyC are marked by gray and blue asterisks, respectively. ***G*,** Characteristic dendritic morphology (left) with spines (right, top) and large MF terminals (right) of the CA3 GC. ***H*,** Axons of the postsynaptic IvyC in the *stratum radiatum* of the CA3. ***I*,** Firing patterns of the cells. ***J*,** Individual data points from all the FF-IN pairs (together with the average data), illustrating the time course of the post-burst potentiation. Download Figure 1-1, PDF file

**Figure 2.**
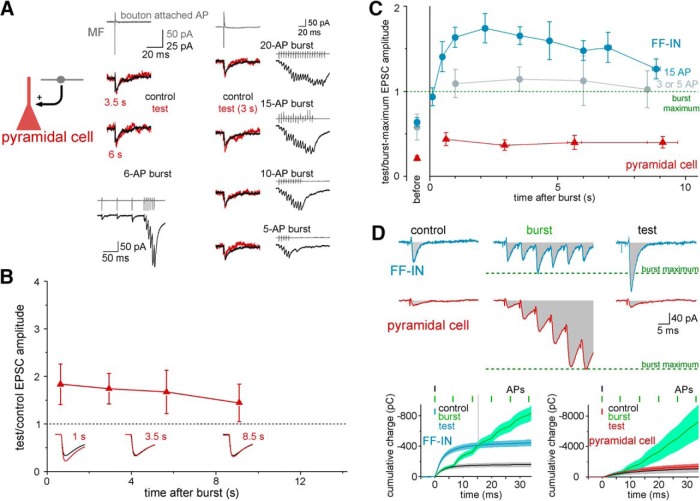
Postsynaptic cell type specificity of postburst potentiation of MF-EPSCs. ***A***, Single presynaptic MF bursts, consisting of various numbers of APs, resulted in small postburst potentiation effects on the EPSCs of a representative MF-pyramidal cell pair. Traces represent presynaptic MF terminal APs in bouton-attached recordings (gray) and the postsynaptic responses before and after single burst. Bursts and postsynaptic responses (including preburst control and postburst test EPSCs, and during-burst responses) are shown next to the corresponding traces. ***B***, Time course of the effects of single, brief presynaptic bursts (6 or 15 AP, 150 Hz) on subsequent single-AP-evoked MF-EPSCs in postsynaptic CA3 pyramidal cells (*n* = 46 data points from *n* = 12 pairs; compare with Fig. 1*B*). Inset, Example control and test MF responses at different postburst delays. Anatomy of the postsynaptic pyramidal cell and presynaptic DG GC (back-labeled via the MF terminal recording) is shown in Figure 2-1. ***C***, Summary graph showing the amplitude of the postburst test responses in pyramidal cells and FF-INs relative to the compound maximal amplitudes reached during the bursts (dashed line) in the same synaptic inputs (red represents pyramidal cells, *n* = 46 data points from *n* = 12 pairs; blue represents FF-INs with 15 AP bursts; light blue represents FF-INs with short, 3 or 5 AP bursts, *n* = 10 pairs; the same responses were reanalyzed as in Fig. 1*B*,*C*). The relative amplitudes of the preburst control responses (“before”) are shown separately. ***D***, Comparison of MF-EPSCs before, during, and after single presynaptic MF bursts in a representative FF-IN (blue traces) and pyramidal cell (red). Green dashed lines indicate maximum amplitude of the compound EPSCs during the burst for comparison. Gray areas represent the integral areas (charge transfer). Bottom, Graphs represent the activity- and postsynaptic cell type-dependent differences of the time course of charge transfer.

10.1523/JNEUROSCI.1595-17.2018.f1-2 Figure 1-2 Postsynaptic axo-axonic cells (AAC): Morphological identification and comparison with the pooled data from all FF-INs. ***A*,** Control and post-burst test MF-EPSCs from an example CA3 GC-AAC pair, 3.5 seconds after the burst. ***B*,** Comparison of the amplification of the MF responses from AAC pairs (n = 10) with the pooled FF-IN data (see **Fig. 1B**). The relative post-burst EPSC amplitudes are shown as in **Fig. 1B** (i.e., control relative amplitudes are 1, dashed line). The insets show the control (before burst) and three different delays after single bursts. ***C*,** Axons of the postsynaptic AAC at the border of *strata pyramidale and oriens*. The MF that originated from the presynaptic CA3 GC is visible at the border of strata lucidum and pyramidale. The inset shows the fast-spiking properties of the postsynaptic AAC. ***D*,** Immunolabeling for PV and SATB1 (negative) of the postsynaptic AAC (Viney et al., 2013). ***E*,** Firing pattern and *stratum radiatum* dendrites of the presynaptic CA3 GC. Download Figure 1-2, PDF file

**Figure 3.**
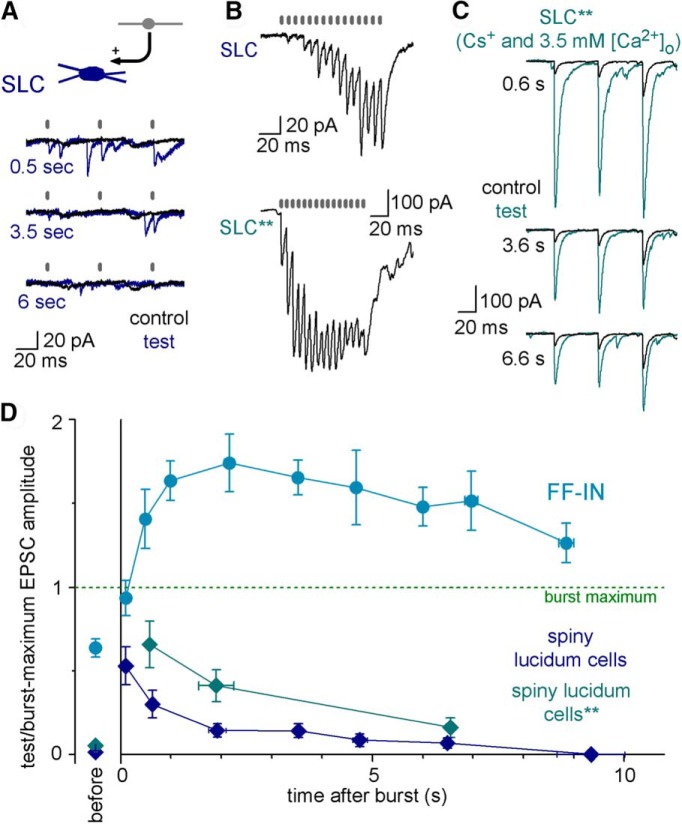
Effects of single MF bursts on the unitary MF responses evoked in SLCs, which are GABAergic but are not considered to be FF-INs. ***A***, Control and postburst test MF-EPSCs from an example CA3 GC-SLC pair at three different time points after single presynaptic bursts. Gray bars represent APs, often weak responses among the frequent spontaneous events. ***B***, The MF responses of two SLCs during the burst (15 APS at 150 Hz) in standard solutions (top) and in the presence of presynaptic intracellular CsCl (by replacing 40 mm KCl) and 3.5 mm extracellular Ca^2+^ level (bottom, **). ***C***, A representative experiment, in which reliable synaptic transmission was obtained in a CA3 GC-to-SLC connection by the above modifications in the recording conditions. ***D***, Summary graph showing the amplitude of the postburst responses in SLCs relative to the compound maximal amplitudes during the bursts. Dark blue represents SLC with standard presynaptic recording (*n* = 61 data points from *n* = 20 pairs). Gray represents SLC with artificially elevated MF release (*n* = 15 data points from *n* = 5 pairs). Blue curve indicates data for FF-INs replotted from Figure 2*C* to highlight the robust differences between the two GABAergic cell groups. The relative amplitudes of the control responses before the bursts are shown separately. For the anatomy of the presynaptic and postsynaptic cells, see Figure 3-1.

10.1523/JNEUROSCI.1595-17.2018.f1-3 Figure 1-3 Postsynaptic CCK-expressing interneurons (CCK+IN): Morphological identification and comparison with the pooled data from all FF-INs. ***A*,** Control and post-burst test MF-EPSCs from an example CA3 GC-CCK+IN pair 4.5 seconds after the burst. ***B*,** Comparison of the amplification of the MF responses from CCK+IN pairs (n = 8) with the pooled FF-IN data (see Fig. 1B). The relative post-burst EPSC amplitudes are shown as in **Fig. 1B** (i.e., control relative amplitudes are 1, dashed line). The insets show the control (before burst, black) and test responses (blue) at four different delays after the single bursts. ***C*,** The presynaptic CA3 GC and the postsynaptic CCK+IN (a basket cell) are highlighted by gray and blue asterisks, respectively. ***D*,** Immunolabeling for CCK and SATB1 (negative) of the postsynaptic cell. ***E*,** Dendrites of the presynaptic CA3 GC. ***F*,** Firing patterns of the pre- and postsynaptic cells. Download Figure 1-3, PDF file

**Figure 4.**
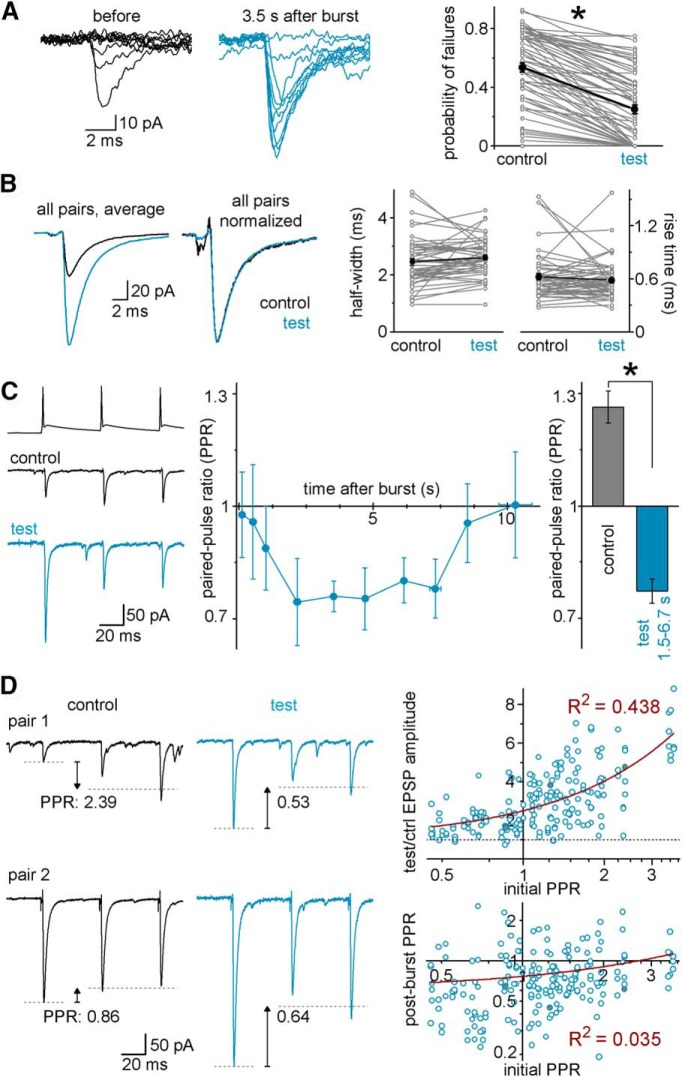
Presynaptic origin of the burst-induced potentiation in FF-INs. ***A***, The probability of synaptic response failures decreases after single presynaptic MF bursts. Representative traces of a CA3 GC-CCK^+^ basket cell connection before (black) and after (blue) single bursts (10 traces from each condition). Right, Connected gray symbols represent the failure rates in individual pairs with identified FF-INs (*n* = 68 pairs) during control and test MF stimuli (1.5–6.7 s postburst delays). Black represents the average data from all FF-IN connections. The failure rates were analyzed from at least 10 trials for each condition. ***B***, Actual and normalized average EPSCs evoked in FF-INs (*n* = 51 pairs) by single MF APs before and 1.5–6.7 s after single presynaptic bursts. The events were aligned to the presynaptic AP peak. Graphs represent the half-width and 10%–90% rise times of the MF responses in individual FF-INs (gray) and their average (black), which were similar before and after the bursts. ***C***, Example traces demonstrating changes in short-term plasticity of the MF-EPSCs in an IvyC evoked by 3 APs before and 6 s after bursts (control PPR: 1.26; test PPR: 0.45). Summary plot shows data from all in identified postsynaptic FF-INs; the PPR (calculated as the ratio of the average of the second and third amplitudes to the first amplitude) was low during the time when the EPSCs were potentiated. Summary bar graphs represent PPRs of all FF-IN connections before (control) and 1.5–6.7 s after the burst (test). ***D***, Example traces: from IvyC MF pairs; note the similarity of test PPRs (at 6 s postburst) despite the different control PPRs. Right panels: Top, Correlation between the control (initial) PPR and the magnitude of potentiation (linear fit, maroon line; note the logarithmic scaling of the PPR axes). Bottom, Independence of postburst test PPR of the control PPR. Each circle represents individual test responses from identified FF-IN pairs. Circles with gray fillings indicate the representative pairs illustrated in ***C*** and ***D***. * marks significant difference between control and test responses (see text for details).

10.1523/JNEUROSCI.1595-17.2018.f1-4 Figure 1-4 Postsynaptic PV-expressing basket cells (PV+BC): Morphological identification and comparison with the pooled data from all FF-INs. ***A*,** Control and post-burst test MF-EPSCs from an example CA3 GC-PV+BC pair 5 seconds after the burst. ***B*,** Comparison of the post-burst potentiation of the MF responses from PV+BC pairs (n = 10) with the pooled FF-IN data (see **Fig. 1B**). The relative post-burst EPSC amplitudes are shown as in **Fig. 1B** (i.e., control relative amplitudes are 1, dashed line). ***C*,** Basket axons, dendrites and firing pattern of the postsynaptic PV+BC. ***D*,** Firing pattern of the presynaptic CA3 GC. ***E*,** Immunolabeling for PV in the dendrites of the postsynaptic cell. Download Figure 1-4, PDF file

In total, we tested the effects of single bursts (in our standard protocol with 15 AP at 150 Hz) in 78 connected pairs in which the postsynaptic interneurons were identified as belonging to the previously reported feedforward category (FF-IN), including ivy cells (IvyCs, *n* = 55 pairs), axo-axonic cells (AACs, *n* = 10 pairs), PV-expressing, fast-spiking basket cells (PV+BCs, *n* = 5 pairs), and regular-spiking CCK-expressing interneurons (CCK+INs, *n* = 8 pairs, including *n* = 3 unequivocally identified CCK basket cells). The differences in the numbers of tested pairs reflect neither the occurrence of the cell types nor the probability of connectivity from GCs (Szabadics and Soltesz, 2009). The basal properties of MF-EPSCs onto FF-INs and their short-term plasticity, ranging between slight facilitation and slight depression (see below), were consistent with previous results (Tóth and McBain, 1998; Tóth et al., 2000; Szabadics and Soltesz, 2009; Torborg et al., 2010). The postsynaptic cells were recorded in voltage-clamp mode, minimizing confounds from spiking activity of the recorded postsynaptic cells to the measured changes.

### Seconds-long potentiation of MF-EPSCs in FF-INs after single MF bursts

First, we studied the postburst consequences of single MF bursts consisting of 15 APs in 100 ms (150 Hz). Such presynaptic AP bursts had surprisingly large effects on the MF-EPSCs in FF-INs, resulting in a tripling of the amplitudes of the test compared with the control responses for several seconds after the burst (Fig. 1*A*,*B*; Figs. 1-1, 1-2, 1-3, and 1-4). Specifically, the effect of the bursts in each pair (*n* = 78 pairs) was examined at 3 or 4 different postburst times for the test responses, and the average increase in test over control responses was 3.09 ± 0.13-fold between 1.5 and 6.7 s after the burst (control response: −43.8 ± 4.6 pA; test: −109 ± 8.6 pA; *p* = 5 × 10^−36^, *t*_(153)_ = 16.58, paired *t* test, total number of postburst test responses at various postburst times, *n* = 154; Fig. 1-1).

As the plot in Figure 1*B* indicates, the burst-induced increase in the MF-EPSCs in FF-INs was not instantaneous, and it gradually developed during the first second after presynaptic burst. After this initial increase, the burst-induced MF-EPSCs remained at a similarly enhanced level for about 8 s (comparison of the responses between 0.8 and 8 s after the burst, in 6 bins by one-way ANOVA: *p* = 0.54, *F*_(5,198,203)_ = 0.814), before the MF-EPSC amplitudes returned to baseline, control values (*p* = 0.081, *t*_(5)_ = 2.184, *n* = 6 timings, *t* test, 9.5–13.5 s after burst). It should be noted that the postburst potentiation shown in Figure 1*B* is distinct from the classical post-tetanic potentiation (Alle et al., 2001; Mori et al., 2007) not only because the latter typically requires considerably more intense presynaptic activity than our physiologically inspired burst protocol, but also because of its temporal profile since post-tetanic potentiation is largest immediately after the high-frequency stimulation and then continuously decays back to baseline. Interestingly, despite the specialized roles and synaptic-cellular mechanisms of the various interneurons within hippocampal circuits (Somogyi et al., 2014), the MF bursts had similar effects on all the postsynaptic FF-IN groups that we examined, including IvyCs, AACs, CCK+INs, and PV+BCs (relative increase in test/control responses: 3.15 ± 0.16, 2.88 ± 0.32, 2.98 ± 0.64, and 2.99 ± 0.3-fold amplification, respectively, one-way ANOVA, *p* = 0.91, *F*_(3,150,153)_ = 0.187; Figs. 1-1, 1-2, 1-3, 1-4).

### Robust effects of single bursts consisting of only a few (3–5) APs

Although the results above were obtained using bursts in which the firing frequency represented typical GC burst (Henze et al., 2002; Pernía-Andrade and Jonas, 2014; GoodSmith et al., 2017) and these bursts contained considerably fewer APs than those in previously used paradigms (Alle et al., 2001; Mori et al., 2007), the 15 APs per burst that we used in the experiments described above were still more than what is found in typical GC bursts *in vivo* (2–7 APs) (Henze et al., 2002; Pernía-Andrade and Jonas, 2014). Therefore, we tested the effectiveness of shorter, truly physiological single bursts, aiming to determine the minimal presynaptic activity range that still results in postburst potentiation of MF-EPSCs. In these experiments, the postburst timing of the test MF-EPSC was kept constant (3 s) and the AP number within the burst was systematically varied between 1 and 20 spikes (e.g., effects of 2-5-9 or 1-4-7-10 or 1-9-17, etc.; AP bursts were tested in any given cell pair, for a total of *n* = 99 combinations). As shown in Figure 1*C* (*n* = 28 pairs), even as low as 2–5 APs resulted in measurable postburst potentiation. Single exponential fits to the measured potentiation predicted that the amplification would reach 50% of its maximal value with a theoretical 2.92 AP burst (±5% confidence range: 2.29–3.7 APs; fit *R*^2^ = 0.966, *p* = 3.8 × 10^−10^). A single burst with 5 APs activated 68.9 ± 8% of the maximal potentiation, and 10 AP bursts increased this value to 90.1 ± 10.2%. Control pulses alone (i.e., omitting the bursts from the protocol) did not evoke potentiation (1.05 ± 0.12; Fig. 1*C*, 0 AP burst point). These data demonstrate that physiological GC AP bursts are well within the dynamic range for the induction of this form of MF plasticity in FF-INs. Interestingly, the effects of shorter bursts also lasted for several seconds (Fig. 1*C*, inset).

Next, to explore the frequency-dependency of the single burst-induced potentiation, we evoked 8 presynaptic APs at 20, 40, 80, and 160 Hz in the same FF-IN-pairs (*n* = 8 pairs; Fig. 1*D*). These presynaptic stimulation protocols did not include preburst control pulses, and the postburst amplitudes were compared with the first responses during the bursts. Interestingly, the amplification showed only a moderate frequency dependence (linear fit, *R*^2^ = 0.769, *p* = 0.08).

Finally, we stimulated presynaptic GCs using bursts that had been observed in *in vivo* recordings previously by other laboratories (Henze et al., 2002; Pernía-Andrade and Jonas, 2014; Diamantaki et al., 2016). These bursts included 4–6 APs with average firing from 100 to 175 Hz. The timing of the spikes within the burst either precisely replicated the *in vivo* data (pattern 1) (Henze et al., 2002) or, if the exact timing of each AP within the actually observed bursts was not available, the bursts of spikes were patterned to reflect the previously reported average frequencies and jitter by considering the approximate frequency-adaptation during bursts: pattern 2 (Pernía-Andrade and Jonas, 2014) and pattern 3 (Diamantaki et al., 2016) (Fig. 1*E*, inset). These *in vivo* bursts and the burst-protocol that we used above (6 APs at 150 Hz) evoked similar potentiation in the same FF-IN-pairs (Fig. 1*E*; *n* = 8 pairs, 3 s after burst, *p* = 0.82, *F*_(3,21)_ = 0.306, one-way repeated measure ANOVA, sphericity assumed).

For the subsequent experiments described below, unless specifically stated otherwise, the more robust 15 AP presynaptic bursts were used to enable us to investigate various aspects and mechanisms of postburst potentiation in FF-INs.

### Postsynaptic cell type specificity of the postburst MF-EPSC potentiation

Next, we tested whether MF inputs to other postsynaptic cell types in the CA3 network, including pyramidal cells and non-FF-IN GABA cells, also exhibit strong potentiation after a single short MF burst. In CA3 pyramidal cells, the postburst test responses were increased compared with the preburst controls (1.72 ± 0.26-fold increase, *n* = 12 pairs, total number of postburst test responses in all pairs at various postburst times: *n* = 25; *p* = 0.01, *t*_(24)_ = 2.798; Fig. 2*A*,*B*), but the potentiation was less robust than the tripling of MF-EPSCS observed in FF-INs (compare Fig. 2*B* with Fig. 1*B*; the data in Fig. 2*B* were obtained using either 6 or 15 AP bursts because, in 4 of the tested pyramidal cell pairs, the 15 AP presynaptic MF bursts resulted in spiking of the postsynaptic pyramidal cell even in voltage clamp; control experiments showed that the postburst potentiation in pyramidal cells remained smaller than in interneurons even after 20 AP bursts; Fig. 2*A*). The difference in the nature of the postburst potentiation between the two cell groups was especially striking when the increase in the test response was expressed with respect to the magnitude of the short-term facilitation of the MF-EPSCs taking place during the bursts. As shown in Figure 2*C*, *D*, when normalized to the maximum responses during the burst, the postburst test responses were significantly larger in the FF-INs compared with the pyramidal cells. These results suggest that the postsynaptic responses in CA3 pyramidal cells are more sensitive to the bursts, while they are relatively more affected during the postburst period in FF-INs.

10.1523/JNEUROSCI.1595-17.2018.f2-1 Figure 2-1 Postsynaptic pyramidal cells: Morphological identification. ***A*,** Two recorded pyramidal cells within the CA3, from which the left cell (asterisk) was the postsynaptic partner in the connections shown in **Fig. 2A**. The insets show the cell body of the postsynaptic pyramidal cell from the neighboring section and the firing pattern of the postsynaptic pyramidal cell. ***B*,** Following the cell-attached presynaptic terminal recording, the presynaptic MF terminal was loaded with biocytin in whole-cell mode, enabling the visualization of the parent GC soma in the DG and typical large MF terminals along its axon (insets). Download Figure 2-1, PDF file

Because the postburst MF-EPSC potentiation was not different between axo-axonic, PV and CCK basket, and ivy cells, we next examined whether the similarity of the effects of single MF bursts extended to non-FF-IN interneurons. The SLCs have negligible local, within-CA3 axons and thus do not participate in feedback or feedforward inhibition but comprise a part of the hippocampo-septal GABAergic projection (Gulyás et al., 1992; Spruston et al., 1997; Jinno et al., 2007). Interestingly, despite the fact that SLCs receive excitatory inputs almost exclusively from MFs (Wittner et al., 2006), the MF synapses on SLCs have extremely low initial release probability, which increases only after sustained presynaptic activity (Szabadics and Soltesz, 2009; Szabadics et al., 2010). Our paired recordings showed that MF synaptic release onto SLCs was increased shortly after the 15 AP MF bursts (Fig. 3*A*,*D*). However, in contrast to FF-INs, the amplitudes of the postburst test MF-EPSCs remained small compared with the compound EPSC amplitudes evoked during the bursts and quickly returned to control baseline (Fig. 3). Thus, the bursts were not able to induce the sustained, seconds-long potentiation of MF-EPSCs in SLCs. Importantly, similarly small burst effects were observed, even when the unusually low control synaptic release probability of the MF-SLC connections (Fig. 3*B*) was artificially increased (Fig. 3*C*,*D*), suggesting that the postsynaptic cell type specificity of the amplification was not simply a consequence of the different initial release probabilities of the MF inputs onto the SLCs and FF-INs.

10.1523/JNEUROSCI.1595-17.2018.f3-1 Figure 3-1 Postsynaptic spiny lucidum cells (SLC): Morphological identification. ***A*,** Firing patterns of the presynaptic CA3 GC and postsynaptic SLC in **Fig. 3A. *B*,** Dendritic morphology of the two cells at low magnification. ***C*,** Dendritic spines of the postsynaptic SLC within the *stratum lucidum*. ***D*,** Immunolabeling of the postsynaptic SLC for somatostatin and CCK (negative). ***E*,** Immunolabeling for somatostatin in the postsynaptic SLC from the pair in **Fig. 3C. *F*,** Firing patterns of the presynaptic CA3 GC and the postsynaptic SLC in Fig. 3C. ***G*,** Nomarski DIC image of the DAB-stained presynaptic CA3 GC and postsynaptic SLC. The inset shows the spiny dendrites of the SLC. Download Figure 3-1, PDF file

### Presynaptic origin of the burst-induced sustained amplification in FF-INs

To better understand the underlying synaptic mechanisms of the single-burst-induced amplification, we tested whether the amplification of the MF-responses in FF-INs was initiated by presynaptic or postsynaptic changes. The probability of failures decreased after single bursts (from 53.3 ± 3% to 25.0 ± 3%; *p* = 3.5 × 10^−20^, *t*_(67)_ = 13.12, *n* = 68 FF-IN pairs, paired *t* test; Fig. 4*A*), consistent with presynaptic changes. The rise times and half-widths of the postburst test MF-EPSCs remained unchanged compared with preburst control (625 ± 38 μs vs 583 ± 31 μs, *p* = 0.25, *t*_(50)_ = 1.155; 2.46 ± 0.12 ms vs 2.61 ± 0.09 ms, *p* = 0.13, *t*_(51)_ = −1.556, paired *t* test; Fig. 4*B*). Comparison of the short-term plasticity of the control and test responses evoked using 3 MF APs at 20 Hz before and after the burst was also consistent with a presynaptic locus for the postburst MF-EPSC potentiation. Specifically, the PPR decreased after the burst and remained small (i.e., more pronounced depression) for several seconds (Fig. 4*C*), similar to the time course of the burst-induced MF-EPSC potentiation in Figure 1*B*.

The PPR of the control (initial, preburst) MF responses varied over a relatively wide range across FF-IN pairs (even within the same types) under baseline conditions. The control PPR showed a correlation with the magnitude of the potentiation (*R*^2^ = 0.438, slope: 1.54 ± 0.12, *F*_(1,196)_ = 154.8, *p* < 10^−20^), indicating that, for connections whose release probability was initially low (i.e., high PPR), single bursts led to larger potentiation, whereas the relative potentiation of the more reliable connections was rather small (Fig. 4*D*). The postburst test PPRs did not correlate with the initial control PPRs of the same connections (*R*^2^ = 0.035, slope: 0.139 ± 0.048, *F*_(1,196)_ = 8.227, *p* = 0.0046), primarily because the PPRs after the bursts were invariably low (i.e., highly reliable, depressing responses), suggesting that single bursts result in a high-release state in MF-FF-IN synapses regardless of their initial reliability.

### Investigations into plasticity mechanisms

**Figure 5.**
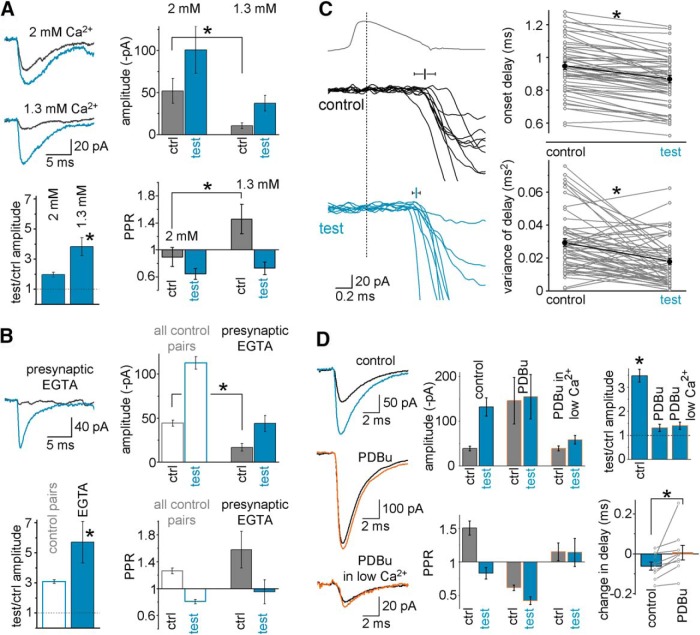
Investigations into mechanisms of postburst plasticity in FF-INs. ***A***, Persistence of the postburst potentiation in lower (1.3 mm; allegedly physiologically more relevant) extracellular Ca^2+^ levels. Example traces are shown from a CA3 GC-IvyC pair in which the effects of single presynaptic bursts were first tested in the presence of standard 2 mm Ca^2+^ concentrations and then in 1.3 mm Ca^2+^ (preburst control responses: black; test responses: blue, 3.6 s after burst). Bar graphs represent average data from all pairs (*n* = 8); the lower Ca^2+^ concentration decreased the response amplitudes and increased the PPRs, but the potentiation persisted. ***B***, Example traces from a CA3 GC-AAC pair in which the effects of single presynaptic bursts were tested while the presynaptic CA3 GC was recorded with 1 mm intracellular EGTA to chelate Ca^2+^. Bar graphs represent average data from all presynaptic EGTA pairs (*n* = 8). Presynaptic EGTA strongly decreased the control responses, but the burst-induced potentiation persisted. ***C***, Example traces illustrate the accelerated and more precise release before and after single presynaptic bursts from the same pair; average presynaptic APs and individual EPSCs are shown; mean and variance of the delay are also indicated, with error bars; failures were excluded for clarity. Right panels, Plots of the mean and variance values of the synaptic onset delay in each MF-FF-IN pair before and after single bursts (connected gray symbols) and their average (black). ***D***, The DAG analog phorbol ester PDBu (1 μm), which promotes vesicle priming, increased MF-EPSC amplitude before bursts (note the different scale bars) and prevented burst-induced potentiation in a representative pair. Subsequent reduction of the release probability following the application of decreased extracellular Ca^2+^ (1 mm) in the PDBu-containing perfusing solution significantly reduced the average amplitudes, but burst-induced amplification remained negligible in the same pair. Summary bar graphs show that bursts were no longer effective in eliciting potentiation in the same pairs in the presence of PDBu regardless of the release probability; furthermore, PDBu also prevented the burst-induced decrease in the delay of the responses (bottom right). Symbols represent changes in synaptic delays in control conditions and in the presence of PDBu. Bars represent average data. * marks significant difference.

To characterize postburst plasticity in FF-INs further, we first explored whether the postburst potentiation at unitary MF-FF-IN synapses was present at a lower extracellular Ca^2+^ concentration (1.3 mm) that may be more physiological (Rancz et al., 2007; Lorteije et al., 2009) than the one used in the standard recording solution (2 mm) in the above-described experiments. In 1.3 mm extracellular Ca^2+^ (Mg^2+^ concentration was kept unchanged at 2 mm), as expected, the control response amplitude decreased and PPR increased (*n* = 8 pairs, *p* = 0.012, *t*_(7)_ = −3.38 and 0.011, *t*_(7)_ = −1.97, respectively, paired *t* test), but the postburst potentiation of the MF-FF-IN inputs not only persisted but increased (from 1.81 ± 0.16 test/control amplitudes to 3.55 ± 0.48 in the same pairs with normal and low Ca^2+^ conditions, respectively, *p* = 0.002, *t*_(12)_ = −3.862, paired *t* test; Fig. 5*A*). The apparently larger postburst potentiation in lower extracellular Ca^2+^ was due to a larger decrease of the control EPSCs compared with the postburst EPSCs (−76.4 ± 1.9% vs −54.3 ± 5.8%, *p* = 0.0017, *t*_(12)_ = −4.034, paired *t* test), consistent with a presynaptic origin for the plasticity. Next, we tested the effects of presynaptic Ca^2+^ chelation on postburst plasticity. Presynaptic loading of the Ca^2+^ chelator EGTA (0.5–2.5 mm; the final concentration in the presynaptic terminal was unknown, as the CA3 GC somatic recording site to presynaptic terminal distance varied) (Szabadics et al., 2010) strongly reduced the control response (*p* = 4 × 10^−4^, *t*_(15.03)_ = 4.615, paired *t* test) but did not prevent the single-burst-induced amplification (*p* = 0.0039, *t*_(15)_ = 3.409, *t* test; Fig. 5*B*). Therefore, postburst potentiation at MF-FF-IN synapses persisted at physiological extracellular Ca^2+^ levels and was consistent with the data shown in Figure 4*D*. In addition, consistent with the results of the 1.3 mm Ca^2+^ and EGTA experiments, when the extracellular Ca^2+^ concentration was elevated to 5 mm, the baseline responses increased by 178 ± 33%, whereas the effect on postburst EPSPs was smaller at 104 ± 28% (*p* = 0.012, *t*_(14)_ = 2.888, paired *t* test, data not shown).

Next, we investigated whether the delay between the presynaptic spike and the onset of the postsynaptic response in FF-INs was altered by the single bursts of MF APs. Our analysis showed that the delay of the test responses was significantly shorter than the delay of the control responses in MF-FF-IN pairs (control: 0.949 ± 0.023 ms; test: 0.868 ± 0.023 ms, *n* = 57 responses, *p* = 9 × 10^−12^, *t*_(56)_ = 8.565, paired *t* test; Fig. 5*C*, top). Furthermore, the variance of the delays was also significantly decreased (from 0.0294 ± 0.0023 ms^2^ to 0.018 ± 0.0018 ms^2^, *p* = 3 × 10^−5^, *t*_(56)_ = 4.544; Fig. 5*C*, bottom; the CV of the delay also changed: from 0.176 ± 0.008 to 0.145 ± 0.008, *p* = 4.5 × 10^−4^, *t*_(56)_ = 3.729). Therefore, the single, brief burst of MF APs results in accelerated, more precise, and more reliable release.

In subsequent experiments, we tested whether the burst-induced plasticity could be occluded by the phorbol ester PDBu, thought to promote vesicle priming and saturate the capacity of the release machinery (Rhee et al., 2002; Lou et al., 2008; Fioravante et al., 2014; Taschenberger et al., 2016). PDBu (1 μm) increased the control response amplitude before the bursts (from −39.6 ± 5.3 pA to −146.3 ± 52.2 pA, *p* = 0.011, *t*_(12)_ = 2.646) and decreased the PPR (from 1.51 ± 0.10 to 0.61 ± 0.04, *p* = 0.0004, *t*_(12)_ = 4.462, *t* test), and there was no significant burst-induced potentiation of the already enhanced MF-EPSCs in FF-INs in the presence of PDBu (test/control amplitude: 1.32 ± 0.15, *p* = 0.21, *t*_(12)_ = 2.11, *t* test; Fig. 5*D*). Furthermore, single MF bursts failed to potentiate the test responses with respect to the preburst control responses in the presence of PDBu, even in reduced extracellular Ca^2+^ (1–1.6 mm), applied to compensate for the amplitude-enhancing effects of PDBu (test/control amplitude: 1.4 ± 0.15, *p* = 0.19, *t*_(15)_ = 2.67; the reduced Ca^2+^ concentration alone would have been expected to increase amplification, see Fig. 5*A*). In addition, application of PDBu also prevented the bursts from accelerating the synaptic release (onset delay of the control and test responses: before PDBu: 1.005 ± 0.069 ms and 0.944 ± 0.082 ms, *p* = 0.017, *t*_(8)_ = 3.01, paired *t* test; in PDBu: 0.96 ± 0.07 ms and 0.967 ± 0.084 ms, *p* = 0.87, *t*_(8)_ = 0.165; ANOVA one-way repeated measure, multivariate test: *p* = 0.048, *F*_(6)_ = 4.851, between-subject effects: *p* = 9.7 × 10^−7^, *F*_(1,8)_ = 177.01; Fig. 5*D*, bottom right). Together, these results suggested occlusion, and thus at least a certain degree of mechanistic convergence, between the burst and PDBu effects, perhaps in the form of enhanced vesicle priming at MF synapses on FF-INs.

**Figure 6.**
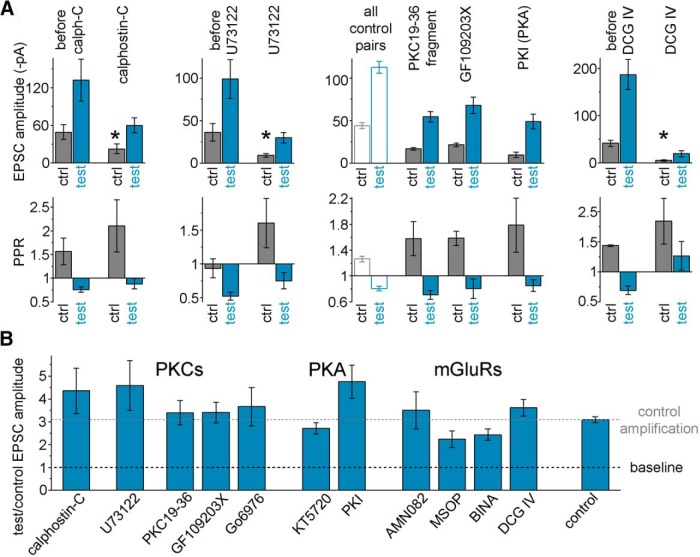
Summary of the pharmacological experiments aimed at probing the potential involvement of various plasticity pathways in the postburst potentiation in FF-INs. ***A***, Calphostin-C (1 μm, *n* = 12) is a PKC (and Munc13) inhibitor that acts on DAG-binding domains. U73122 (2.5 μm, *n* = 8) is a phospholipase C inhibitor. Both inhibitors affected baseline release as indicated by the attenuation of the control response amplitudes indicating the involvement of these pathways in the regulation of release at these synapses, serving as positive internal controls indicating drug effectiveness. PKC19-36 (100 μm, *n* = 7, synthetic autoinhibitory domain, applied intracellularly) and GF109203X (1 μm, *n* = 11, acts on the ATP binding site; slices were preincubated for at least 60 min) are selective PKC inhibitors. The small control amplitudes and large PPRs provided positive internal controls for these inhibitors. The averages of all control responses are also shown for comparison (because these inhibitors required intracellular application and pretreatment). DCG IV is an mGluR2/3 agonist (1 μm, *n* = 7) that selectively inhibits MF responses. * marks significant difference in the control (before burst) EPSC amplitudes in control conditions and after drug application. ***B***, Average amplification at 1.5–6.7 s after single 15 AP bursts in the presence of various pharmacological agents. In addition to the above drugs, the burst-induced amplification of MF-EPSCs is shown in the presence of Go6976 (0.25 μm, *n* = 8, a subtype-selective PKC inhibitor), KT5720 (200 nm, *n* = 10, a PKA inhibitor that works by competing with ATP binding; PKAs are involved in pathways that regulate presynaptic release, including RIM proteins, whose PKA-dependent phosphorylation promotes vesicle priming and PKAs are also involved in posttetanic potentiation mechanisms at MF synapses onto DG inhibitory cells) (Alle et al., 2001), PKI (2.5 μm intracellularly, PKA inhibitory fragment 6–22 amide, that binds to the substrate site) (Hashimotodani et al., 2017), AMN082 (1 μm, *n* = 6, selective mGluR7 agonist), MSOP (150 μm, *n* = 8, selective inhibitor of Group III mGluRs including mGluR7), or BINA (5 μm, *n* = 12, selective positive allosteric modulator of mGluR2). For additional positive control effects, see Results. Postburst potentiation in the absence of these drugs (“control amplification”) is also shown for comparison. None of these pharmacological modifiers of known plasticity pathway components was able to significantly block the postburst potentiation.

We also investigated whether mechanisms implicated in other forms of plasticity at MF synapses were involved in the postburst potentiation described in this paper. PKC and Munc13 proteins are known to be involved in regulating release at many synapses (Betz et al., 1998; Augustin et al., 1999; Wu and Wu, 2001; Rosenmund et al., 2002; Junge et al., 2004; Korogod et al., 2007; Wierda et al., 2007; Fioravante et al., 2011; Chu et al., 2014), including MFs (Alle et al., 2001; Pelkey et al., 2005; Hainmüller et al., 2014). In agreement with these results, various direct and indirect inhibitors of PKC activity (calphostin-C, intracellular PKC19-36, GF109203X, and U73122, the latter inhibits PLCs that generate diacyl-glycerol for PKC functions) were able to decrease the control EPSCs at MF-FF-IN synapses. Specifically, acting as positive controls, the drug application reduced the control EPSC amplitudes (calphostin-C, with respect to predrug: 60.5 ± 27.4%; U73122: to 37.5 ± 17.5%) and increased the PPR (by 0.53 ± 0.55 and 0.67 ± 0.37, respectively; Fig. 6*A*); similarly, in the case of experiments with PKC19-36 fragment and GF109203X (these drugs were preincubated), the EPSC amplitudes were 37.9 ± 3.2% and 48.6 ± 5%, respectively, of the untreated control pairs, and the PPRs were increased by 0.32 ± 0.26 and 0.32 ± 0.11, respectively. In contrast, another type of PKC blocker, Go6976, did not alter the control MF responses (control EPSC amplitude with respect to predrug control: 108.6 ± 55.2%; PPR: 0.24 ± 0.47).

Importantly, if PKC activity were necessary for the burst-induced potentiation, these various PKC inhibitors should have decreased or eliminated the postburst plasticity. However, the postburst potentiation was not affected in the presence of any of these inhibitors (Fig. 6*B*, group of bars under the label “PKCs”), despite the presence of positive control effects for most of the drugs. Because Munc13 proteins share both active domains (which are targeted by calphostin-C, PDBu, and native diacyl-glycerol sources) and synaptic functions with PKCs (promoting release), we cannot exclude the possibility that Munc13s were involved in the positive control effects, but it is also unlikely that they were necessary for the burst-induced potentiation. Similarly, we were unable to block the postburst potentiation in FF-INs using the PKA inhibitors KT5720 and presynaptically loaded PKI6–22 fragment (Fig. 6*B*, bars under the label “PKA”; the lack of an effect on the postburst potentiation was in the presence of positive control effects on the control EPSC amplitudes: KT5720, 51.2 ± 17.8%; and PPR, 0.24 ± 0.25). In addition, neither activation nor inhibition of mGluR7 and mGluR2/3 occluded or eliminated the postburst potentiation (Fig. 6*B*, bars labeled “mGluRs”) (AMN082, control effect on EPSC and PPR, respectively: 70.2 ± 33.7%, 0.51 ± 039; MSOP, 62 ± 21.7%, −0.18 ± 0.17; BINA, 78.6 ± 9.8%, −0.09 ± 0.12; and DCG-IV, 11.3 ± 4%, 0.41 ± 0.38; mGluR7 is specifically expressed at MF synapses on GABAergic cells) (Shigemoto et al., 1997; Pelkey et al., 2008). The fact that none of these pharmacological manipulations of PKC, PKA, and mGluRs was able to significantly modify postburst potentiation (note the remarkably stable level of potentiation in the presence of various drugs in Fig. 6*B*) suggested that the single, brief burst-induced MF plasticity at FF-IN synapses described in this study may involve a potentially noncanonical molecular pathway that will need to be identified in the future.

### Seconds-long potentiation of FF-IN activity in the CA3 network after individual MF bursts

**Figure 7.**
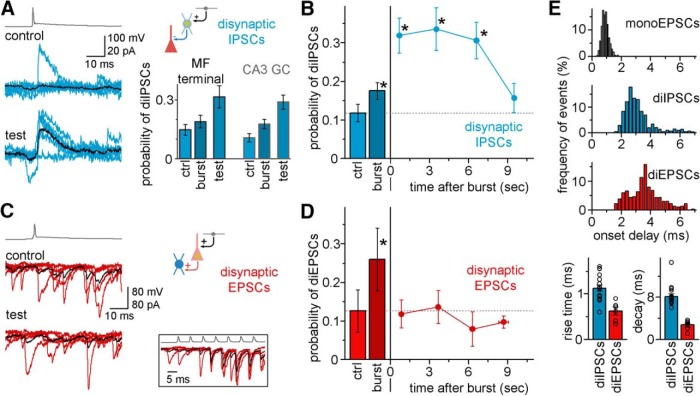
Differential effects of single bursts in individual MFs on diIPSCs and diEPSCs. ***A***, Example traces: Synaptic events in a single randomly chosen CA3 pyramidal cell during the stimulation of a simultaneously recorded single CA3 GC that was not monosynaptically connected to the pyramidal cell; the majority of the outward events are disynaptic GABAergic responses (diIPSCs; at −50 mV using low chloride internal solution in postsynaptic cells), which presumably originate from one or more nonrecorded FF-IN(s); there is increased probability of diIPSC after single bursts in the MF (test). Black average traces represent the increased weight of disynaptic inhibition after the bursts. Right, Bar graphs represent summary data from diIPSC experiments. The bar graphs illustrate two types of diIPSC pairs: one in which the presynaptic MF source was a MF terminal (presumably originating from DG GCs), the other in which the MF source was a somatically recorded CA3 GC. There is a lack of differences in the burst-induced changes in the diIPSCs, suggesting that these two MF recording configurations induce similar burst-dependent effects on the CA3 feedforward inhibitory circuit. ***B***, Summary data indicating that the postburst time course of the increase in the probability of single MF-evoked diIPSCs. There is similarity of the time course to that of the burst-induced potentiation of the MF-EPSCs in FF-INs in Figure 1*B*. The increase in the probability of the diIPSCs during the burst (dark blue) was smaller than it was after the burst, consistent with the FF-IN data in Figure 2*C*. ***C***, Example traces: diEPSC events recorded from a nonidentified fast-spiking interneuron during the activation of a CA3 GC; these diEPSCs presumably reflect the spiking of one or more nonrecorded pyramidal cell(s); there are frequent diEPSCs during the high-frequency bursts (inset), whereas the probability of diEPSCs remained unchanged after the bursts. ***D***, Summary data: burst effects on the probability of diEPSCs evoked by MFs before, during (bars), and after bursts in the same individual MF (average data of 6 pairs, with 15 AP bursts at 150 Hz); note the difference compared with the data in ***B***, and the similarity to the MF-EPSC pyramidal cell data in Figure 2*C*. * mark significant difference in the probability of disynaptic events relative to control probabilities (before burst). ***E***, Distribution of the onset time delays of individual postsynaptic events measured from the time of the presynaptic AP, including monosynaptic EPSCs in postsynaptic FF-INs (gray), diIPSCs in CA3 pyramidal cells (blue), and diEPSCs in GABAergic cells (red). The 10%–90% rise time and decay time constants of the diIPSCs and diEPSCs are shown below. Each data point represents an individual pair.

Our results suggest that FF-INs and pyramidal cells in the CA3 area respond to single MF bursts differently, with weak (strong) short-term facilitation of the unitary EPSCs but strong (weak) postburst potentiation in FF-INs (pyramidal cells) (Fig. 2*B–D*). In other words, the seconds-long MF-EPSC plasticity in FF-INs appear to reflect the recent passage of a MF burst, whereas changes in MF-EPSCs in pyramidal cells preferentially take place during the MF bursts themselves. To test these ideas further, in the final series of experiments, we examined whether a single brief burst of APs in an individual MF is able to increase FF-IN activity in the network by measuring the incidence of diIPSCs in pyramidal cells that were not themselves synaptically connected to the activated MF (for synaptic delays and kinetics of diIPSCs after APs in the MF, see Fig. 7*E*) (Neubrandt et al., 2017). The probability of diIPSCs was modestly but significantly increased in randomly selected pyramidal cells during the bursts of APs in individual MFs (Fig. 7*A*,*B*, bar labeled “burst”; diIPSCs after APs in a single MF: control responses: 11.9 ± 3.4%, during bursts: 18.1 ± 1.9%, *p* = 0.0029, *t*_(23)_ = −3.329, paired *t* test, *n* = 24 pairs of MF and randomly chosen pyramidal cell). In contrast, in the same pairs, the probability of diIPSCs almost tripled for several seconds following the AP burst in the individual MF, with a time course that showed remarkable similarity to the potentiation of unitary MF-EPSCs after bursts (compare Fig. 7*B* with Fig. 1*B*; probability of diIPSCs in response to test pulses 1.5–6.7 s after the bursts: 29.4 ± 3.1%, *p* = 1 × 10^−9^, *t*_(39)_ = −7.944, compared with control preburst responses and *p* = 2 × 10^−4^, *t*_(39)_ = −4.09, compared with diIPSCs during the bursts, paired *t* test). These data indicated that a single burst in an individual MF enhanced the probability of IPSCs for several seconds in a population of pyramidal cells that were not directly connected to the activated MFs, presumably through the involvement of one or more unrecorded FF-IN(s). In addition, these results were similar regardless of whether we evoked bursts in a giant MF terminal or in somatically recorded CA3 GCs (Szabadics et al., 2010) (Fig. 7*A*, bottom right inset, bar graphs; because DG GCs are much more numerous than CA3GCs, the MF terminal recordings must have overwhelmingly come from MFs of DG origin; therefore, these results also showed that the burst-related plasticity in FF-INs was similar with CA3 GC or DG GC inputs). In contrast, when we assessed the effect of single bursts in individual MFs on pyramidal cell activation by recording disynaptic EPSCs from CA3 FF-INs, we found a strong increase in the probability of diEPSCs during the bursts (control: 12.6 ± 5.5%, during burst: 26 ± 8.1%, *n* = 6 pairs, *p* = 0.015, *t*_(5)_ = −3.649, paired *t* test), but no enhancement of the probability of diEPSCs after the bursts (Fig. 7*C*,*D*; probability of diIPSCs after test pulses 1.5–6.7 s following the burst: 10.6 ± 2.9%, *p* = 0.61, *t*_(11)_ = 0.521, paired *t* test). These results supported our hypothesis that pyramidal cells were more sensitive to the bursts, whereas the inhibitory network preferentially represented the recent past of MF burst activity.

## Discussion

### Unusual properties of postburst potentiation at MF-FF-IN synapses

The results in this paper report the existence of a seconds-long, novel form of synaptic plasticity taking place between presynaptic MFs and postsynaptic CA3 FF-INs. A major distinguishing feature of the plasticity is that it could be triggered by single, brief presynaptic burst activity consisting of as few as 3 APs at 150 Hz, as well as by naturally occurring bursts recorded from GCs *in vivo*. The postburst potentiation also had an unusual time course, as it took almost a full second to develop, had no clear maximum, and persisted at a similar level for several seconds. An order of magnitude stronger stimulation paradigm (100 AP, delivered in 2.5 s) applied to single GCs in hippocampal slice cultures have been shown to cause an increase in feedforward inhibition in the CA3 for >10 min (Mori et al., 2004, 2007). Apart from the distinct time course, the latter plasticity sharply differed from the postburst potentiation reported in this paper because it was expressed in GABAergic neurons as well as pyramidal cells (Mori et al., 2007). Similarly, the brief unitary burst-induced potentiation in interneurons reported in the current study also differs from the short-term facilitation and long-term MF plasticity taking place in CA3 pyramidal cells after cooperative stimulation of multiple GCs with spike trains consisting of tens of spikes associated with place field traversals (Gundlfinger et al., 2010). Therefore, our results reveal a novel form of synaptic plasticity evoked by physiologically relevant stimuli (Dobrunz and Stevens, 1999; Gundlfinger et al., 2010).

Interestingly, the cell-type specificity of the MF-induced postburst potentiation had two complementary aspects. On the one hand, it occurred similarly in both perisomatically (e.g., axo-axonic cells, PV+BCs) and dendritically projecting (e.g., ivy cells) FF-INs, despite their distinct intrinsic and synaptic properties, connectivity and local circuit functions (Ruediger et al., 2011; Somogyi et al., 2014). On the other hand, the postburst potentiation was much weaker or nonexistent in SLCs (which do not contribute to feedforward inhibition), highlighting the fact that the plasticity was cell type-specific, even among GABAergic cells. MFs are known to provide monosynaptic innervation to four distinct GABAergic cell types in the CA3, including cells with local (FF-INs, including PV cells, CCK+Ins, and ivy cells) and extrahippocampal (SLC) projections. SLCs are projection cells that mainly target the medial septum, and their primary dendrites are largely restricted to the stratum lucidum (and, to a lesser extent, the stratum pyramidale) (Gulyás et al., 1992; Jinno et al., 2007), with densely placed spines on the dendrites. A curious aspect of these cells is that, although they receive most of their inputs from MFs (Wittner et al., 2006), the existence of monosynaptic MF inputs is often revealed only after >10 APs at 50 Hz, and our experiments showed that SLCs lacked MF postburst potentiation even under artificial conditions (high extracellular Ca^2+^) when the probability of successful EPSCs after presynaptic action potentials was increased. Interestingly, small MF terminals specialized to innervate GABA cells can be either filopodial extensions that emanate from the large MF terminals or en passant boutons. However, it is not known whether the FF-INs and SLCs are selectively innervated by specific small MF types, and it is also unclear whether the observed differences in the MF burst-induced potentiation between the FF-INs and SLCs correlates with the nature of the small MF presynaptic element.

In addition to the within-GABA cell group differences, the cell type specificity of the postburst plasticity was especially apparent when FF-INs and CA3 pyramidal cells were compared. Together, the results indicated a remarkable temporal differentiation of MF potentiation effects at the MF giant terminal to pyramidal cell versus the filopodial and en passant small bouton synapses on FF-INs (Acsády et al., 1998), with CA3 pyramidal cells displaying the well-known frequency facilitation during the bursts, whereas FF-INs exhibiting preferential augmentation of the MF responses for several seconds after the passage of a single brief MF burst. The significantly decreased delay and variability of onset of the MF-EPSCs during the postburst period in the FF-INs indicated accelerated, more precise, and more reliable release as being the key features of the postburst plasticity at MF-FF-IN synapses, perhaps through the involvement of enhanced vesicle priming (as suggested by the apparent convergence of the burst- and PDBu effects) (Rhee et al., 2002; Lou et al., 2008; Fioravante et al., 2014; Taschenberger et al., 2016). Curiously, various pharmacological manipulations of PKC, PKA, and mGluRs (Alle et al., 2001; Pelkey et al., 2005, 2008; Gundlfinger et al., 2010; Hainmüller et al., 2014) did not yield significant inhibition of the postburst potentiation in FF-INs, even in the presence of positive controls, raising the possible involvement of an unusual form of plasticity mechanism that will need to be investigated in the future.

In conclusion, different forms of short-term plasticity mechanisms have been identified as controllers of spike transfer at DG-CA3 connections (Nicoll and Schmitz, 2005; Evstratova and Tóth, 2014; Cherubini and Miles, 2015; Zucca et al., 2017) assisting in pattern completion and pattern separation tasks (Guzowski et al., 2004; Rolls and Treves, 2011). Local feedforward inhibitory circuits have been recognized to play key roles in controlling information transfer at MF-CA3 inputs, including short-term plasticity mechanisms at MF-FF-IN synapses lasting for tens of milliseconds (Szabadics and Soltesz, 2009; Torborg et al., 2010) and a 100-ms-long inhibition mediated by GABA_B_ receptors *in vivo* (Zucca et al., 2017). Acting at considerably longer time-scales, the potentiation of the MF inputs to FF-INs after a single, brief burst of APs in individual MFs reported in the present study was strong enough to cause a robust enhancement of disynaptic IPSCs, even in randomly sampled pyramidal cells that were not themselves monosynaptically connected to the activated individual MF. Therefore, it appears that single physiologically realistic bursts in individual GCs are able to increase feedforward inhibition in a sizeable portion of the CA3 network (e.g., at spatial scales corresponding to a hippocampal lamella partially represented in a hippocampal acute slice). Although recent results demonstrated that GCs typically possess only single place fields and fire at low frequencies (Danielson et al., 2017; GoodSmith et al., 2017; Senzai and Buzsáki, 2017), the sheer numerical abundance of GCs seems to imply that even the relatively sparse occurrence of burst discharges (Diamantaki et al., 2016) in individual GCs may collectively result in the CA3 FF-IN network being placed in a postburst potentiated state most of the time. Alternatively, the ability of solitary brief bursts in single GCs to powerfully enhance inhibition at the DG-CA3 interface in the seconds-long time-scales of interburst intervals *in vivo* may play a role in setting the sensitivity of specific subsets of CA3 ensemble as a function of recent history of GC activity (e.g., during reentry of a recently visited place field where specific GCs fired burst of Aps) (Diamantaki et al., 2016), potentially regulating the contrast between representations of two similar events in CA3 circuits. Future tests of these not necessarily mutually exclusive hypotheses will help to develop a quantitative understanding of the ability of presynaptic GCs to recruit strong feedforward inhibition in populations of CA3 pyramidal cells, likely aided by *in vivo* experiments (Pernía-Andrade and Jonas, 2014; Zucca et al., 2017) in combination with biologically realistic, data-driven large-scale computational network models (Bezaire et al., 2016). Although several aspects of the MF postburst potentiation in CA3 FF-INs will need to be explored further, the results reported in this paper are in general agreement with the notion that bursts represent a unique signal that is distinct from single spikes and even the arithmetically summed effects of single spikes (Lisman, 1997; Kaifosh and Losonczy, 2016).
