# Supplementary material for: Single Bursts of Individual Granule Cells Functionally Rearrange Feedforward Inhibition
Source: J Neurosci. 2018 Feb 14;38(7):1711–24. doi: 10.1523/JNEUROSCI.1595-17.2018 (PMC5815453; doi:10.1523/JNEUROSCI.1595-17.2018)
Supplement: Figure 1-1 [file zns999180525so1.pdf]

Single bursts of individual granule cells functionally rearrange feed-forward inhibition

Máté Neubrandt[1], Viktor János Oláh[1], János Brunner[1], Endre Marosi[1], Ivan Soltesz[2,3], János Szabadics[1]

[1] Institute of Experimental Medicine, Hungarian Academy of Science, Szigony utca 43, Budapest, 1083, Hungary

[2] Department of Neurosurgery,

[3] Stanford Neurosciences Institute, Stanford University, Stanford, CA 94305, USA

Extended Data: Anatomical data for the identification of postsynaptic cells and individual data points

Figure 1-1, 1-2, 1-3, 1-4: postsynaptic FF-INs

Figure 2-1: postsynaptic PC and back-labelled DG GC

Figure 3-1: postsynaptic SLCs

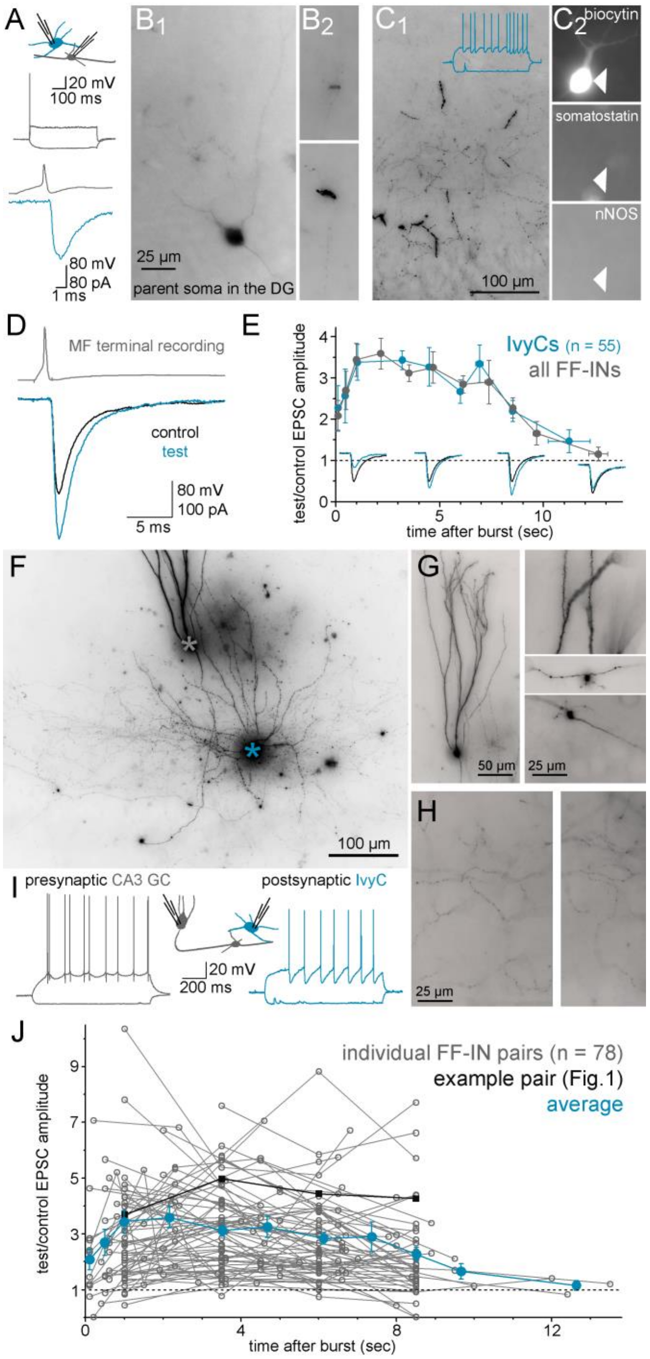

Figure 1-1. Postsynaptic Ivy cells (IvyC): Morphological identification, comparison with the pooled data from all FF-INs and representative MF terminal (presynaptic DG GC) to IvyC and CA3 GC to IvyC pairs. ***A,*** Upper panel: firing pattern of the presynaptic MF terminal; lower: example traces show the presynaptic AP in the MF terminal and the EPSC response in the IvyC. ***B,*** Back-labeled parent GC soma (B_1) in the DG following the recording of the presynaptic MF terminal in the CA3; and MF terminals (B_2) along the recorded axon. ***C,*** Axon morphology (C_1) and immunohistochemical testing (C_2) of the postsynaptic IvyC in the *stratum radiatum* of the CA3. The inset shows the firing pattern of the IvyC. ***D,*** Control and post-burst test MF-EPSCs from the example MF bouton-IvyC pair, 8.4 seconds after the burst (15 AP at 150 Hz). ***E,*** Comparison of the amplification of the MF responses from IvyC pairs (n = 55) with the pooled FF-IN data (see Fig. 1B). The insets show the control (before

burst) and four different delays after the single bursts from the pair shown in panel D. ***F-G***, Identification of the presynaptic CA3 GC and the postsynaptic IvyC shown in **Fig. 1A-B**. ***F,*** The presynaptic CA3 GC and the postsynaptic IvyC are marked by gray and blue asterisks, respectively. ***G,*** Characteristic dendritic morphology (left) with spines (right, top) and large MF terminals (right) of the CA3 GC. ***H,*** Axons of the postsynaptic IvyC in the *stratum radiatum* of the CA3. ***I,*** Firing patterns of the cells. ***J,*** Individual data points from all the FF-IN pairs (together with the average data), illustrating the time course of the post-burst potentiation.

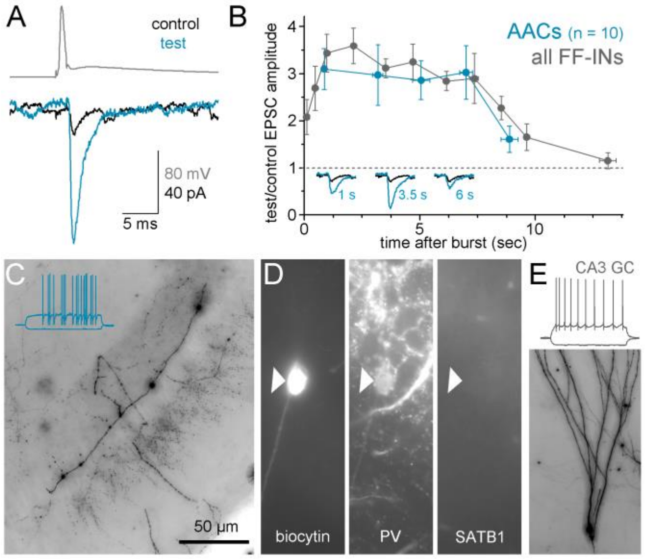

Figure 1-2. Postsynaptic axo-axonic cells (AAC): Morphological identification and comparison with the pooled data from all FF-INs. ***A,*** Control and post-burst test MF-EPSCs from an example CA3 GC-AAC pair, 3.5 seconds after the burst. ***B,*** Comparison of the amplification of the MF responses from AAC pairs (n = 10) with the pooled FF-IN data (see **Fig. 1B**). The relative post-burst EPSC amplitudes are shown as in **Fig. 1B** (i.e., control relative amplitudes are 1, dashed line). The insets show the control (before burst) and three different delays after single bursts. ***C,*** Axons of the postsynaptic AAC at the border of *strata pyramidale and oriens*. The MF that originated from the presynaptic CA3 GC is visible at the border of strata lucidum and pyramidale. The inset shows the fast-spiking properties of the postsynaptic AAC. ***D,*** Immunolabeling for PV and SATB1 (negative) of the postsynaptic AAC (Viney et al., 2013). ***E,*** Firing pattern and *stratum radiatum* dendrites of the presynaptic CA3 GC.

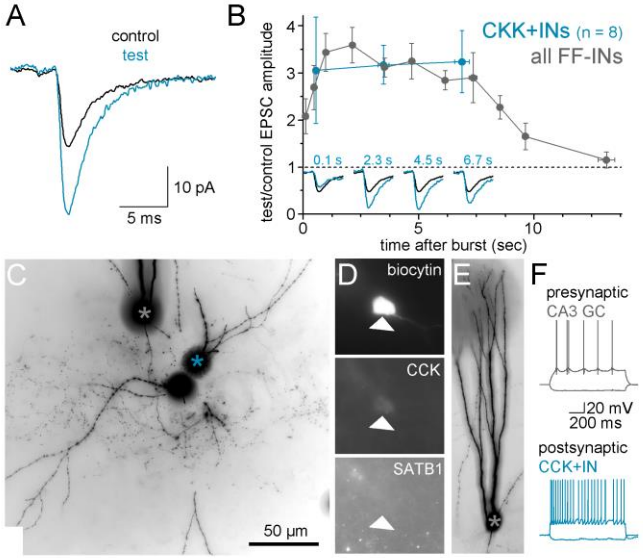

Figure 1-3. Postsynaptic CCK-expressing interneurons (CCK+IN): Morphological identification and comparison with the pooled data from all FF-INs. ***A,*** Control and post-burst test MF-EPSCs from an example CA3 GC-CCK+IN pair 4.5 seconds after the burst. ***B,*** Comparison of the amplification of the MF responses from CCK+IN pairs (n = 8) with the pooled FF-IN data (see Fig. 1B). The relative post-burst EPSC amplitudes are shown as in **Fig. 1B** (i.e., control relative amplitudes are 1, dashed line). The insets show the control (before burst, black) and test responses (blue) at four different delays after the single bursts. ***C,*** The presynaptic CA3 GC and the postsynaptic CCK+IN (a basket cell) are highlighted by gray and blue asterisks, respectively. ***D,*** Immunolabeling for CCK and SATB1 (negative) of the postsynaptic cell. ***E,*** Dendrites of the presynaptic CA3 GC. ***F,*** Firing patterns of the pre- and postsynaptic cells.

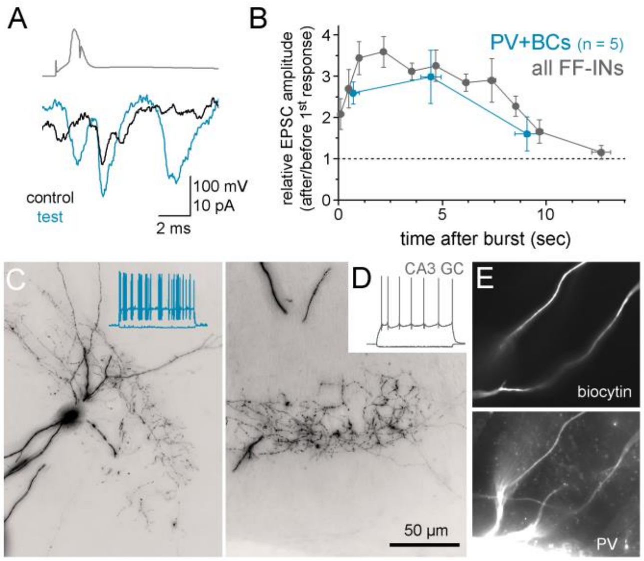

Figure 1-4. Postsynaptic PV-expressing basket cells (PV+BC): Morphological identification and comparison with the pooled data from all FF-INs. ***A,*** Control and post-burst test MF-EPSCs from an example CA3 GC-PV+BC pair 5 seconds after the burst. ***B,*** Comparison of the post-burst potentiation of the MF responses from PV+BC pairs (n = 10) with the pooled FF-IN data (see **Fig. 1B**). The relative post-burst EPSC amplitudes are shown as in **Fig. 1B** (i.e., control relative amplitudes are 1, dashed line). ***C,*** Basket axons, dendrites and firing pattern of the postsynaptic PV+BC. ***D,*** Firing pattern of the presynaptic CA3 GC. ***E,*** Immunolabeling for PV in the dendrites of the postsynaptic cell.

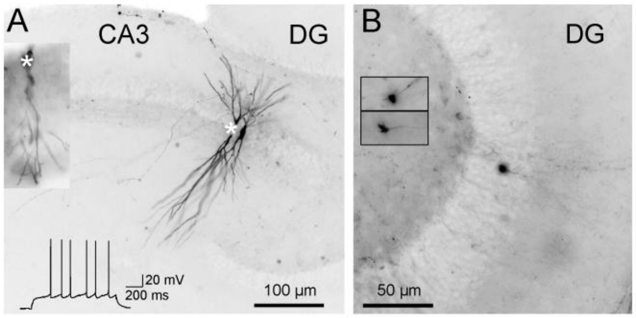

Figure 2-1. Postsynaptic pyramidal cells: Morphological identification. ***A,*** Two recorded pyramidal cells within the CA3, from which the left cell (asterisk) was the postsynaptic partner in the connections shown in **Fig. 2A**. The insets show the cell body of the postsynaptic pyramidal cell from the neighboring section and the firing pattern of the postsynaptic pyramidal cell. ***B,*** Following the cell-attached presynaptic terminal recording, the presynaptic MF terminal was loaded with biocytin in whole-cell mode, enabling the visualization of the parent GC soma in the DG and typical large MF terminals along its axon (insets).

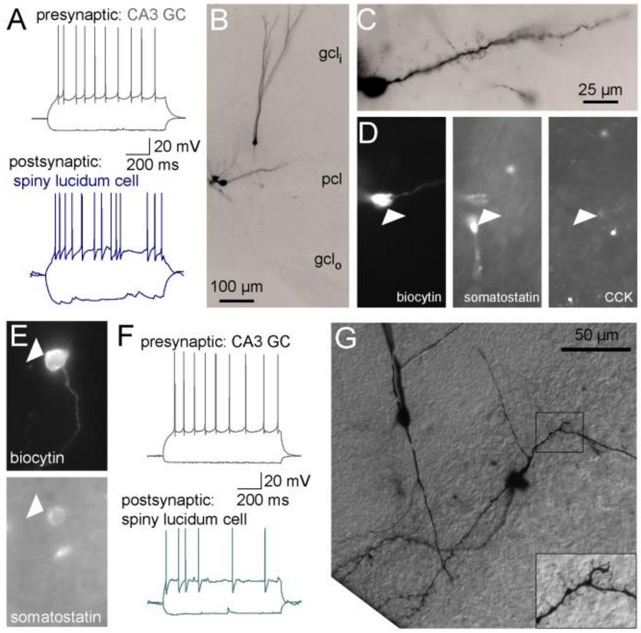

Figure 3-1. Postsynaptic spiny lucidum cells (SLC): Morphological identification. ***A,*** Firing patterns of the presynaptic CA3 GC and postsynaptic SLC in **Fig. 3A**. ***B,*** Dendritic morphology of the two cells at low magnification. ***C,*** Dendritic spines of the postsynaptic SLC within the *stratum lucidum*. ***D,*** Immunolabeling of the postsynaptic SLC for somatostatin and CCK (negative). ***E,*** Immunolabeling for somatostatin in the postsynaptic SLC from the pair in **Fig. 3C**. ***F,*** Firing patterns of the presynaptic CA3 GC and the postsynaptic SLC in Fig. 3C. ***G,*** Nomarski DIC image of the DAB-stained presynaptic CA3 GC and postsynaptic SLC. The inset shows the spiny dendrites of the SLC.

Reference in Extended Data (this reference was also cited in the main text)

Viney, T.J., Lasztoczi, B., Katona, L., Crump, M.G., Tukker, J.J., Klausberger, T., and Somogyi, P. (2013). Network state-dependent inhibition of identified hippocampal CA3 axo-axonic cells in vivo. Nat Neurosci *16*, 1802-1811.
